# 2-year survival of patients undergoing mild hypothermia treatment after ventricular fibrillation cardiac arrest is significantly improved compared to historical controls

**DOI:** 10.1186/1757-7241-18-2

**Published:** 2010-01-08

**Authors:** Christian Storm, Jens Nee, Anne Krueger, Joerg C Schefold, Dietrich Hasper

**Affiliations:** 1 Charité Universitätsmedizin Berlin, Campus Virchow-Klinikum, Department of Nephrology and Medical Intensive Care, Augustenburger Platz 1, 13353 Berlin, Germany

## Abstract

**Background:**

Therapeutic hypothermia has been proven to be effective in improving neurological outcome in patients after cardiac arrest due to ventricular fibrillation (VF). Data concerning the effect of hypothermia treatment on long-term survival however is limited.

**Materials and methods:**

Clinical and outcome data of 107 consecutive patients undergoing therapeutic hypothermia after cardiac arrest due to VF were compared with 98 historical controls. Neurological outcome was assessed at ICU discharge according to the Pittsburgh cerebral performance category (CPC). A Kaplan-Meier analysis of follow-up data concerning mortality after 24 months as well as a Cox-regression to adjust for confounders were calculated.

**Results:**

Neurological outcome significantly improved after mild hypothermia treatment (hypothermia group CPC 1-2 59.8%, control group CPC 1-2 24.5%; p < 0.01). In Kaplan-Meier survival analysis hypothermia treatment was also associated with significantly improved 2-year probability for survival (hypothermia 55% vs. control 34%; p = 0.029). Cox-regression analysis revealed hypothermia treatment (p = 0.031) and age (p = 0.013) as independent predictors of 24-month survival.

**Conclusions:**

Our study demonstrates that the early survival benefit seen with therapeutic hypothermia persists after two years. This strongly supports adherence to current recommendations regarding postresuscitation care for all patients after cardiac arrest due to VF and maybe other rhythms as well.

## Introduction

Patients surviving cardiac arrest still have a poor prognosis with regard to both mortality and neurological outcome. Current guidelines recommend mild hypothermia treatment after cardiac arrest due to ventricular fibrillation (VF) as well as for other initial rhythms[1,2]. These recommendations are based on published data demonstrating a significantly improved outcome with therapeutic hypothermia, especially after VF cardiac arrest. In these studies follow-up time ranged between 3 and 6 months[3,4].

Recent studies show that neurological performance does not change markedly from the time of ICU discharge to six months after cardiac arrest in the majority of patients [5]. In a few patients functional outcomes improved over time, while deterioration was rarely seen [6]. Mortality of course is also an important factor when evaluating the long-term effects of therapeutic hypothermia. Therefore we have analyzed the probability of 2-year survival in a cohort of patients undergoing therapeutic hypothermia and compared these data to historical controls.

## Materials and methods

The study protocol was approved by the local ethics committee on human research. Between 2005 and 2007 a total of 107 patients were admitted consecutively to our MICU after in-hospital (IHCA) or out-of-hospital cardiac arrest (OHCA). Hypothermia treatment was applied to all survivors after cardiac arrest (n = 107) for 24 hours. A historical control group treated prior to the implementation of hypothermia protocols was identified (n = 98 patients admitted to our MICU between 2002 and 2004 after cardiac arrest). Detailed characteristics for the study population are given in Table 1. All patients received standard post resuscitation care which did not undergo notable changes during the observation period except for the application of therapeutic hypothermia. In the treatment group hypothermia was maintained for 24 hours using a surface cooling device (ArcticSun2000^® ^Medivance, USA).

**Table 1:** Baseline characteristics of the study population (n = 205)

Variable	Control (n = 98)	Hypothermia (n = 107)	p-Value
Age (years)	64.5 (59.61-64.90)	60.5 (57.40-62.22)	0.13
Female sex-no./total no.(%)	26/98 (26.5)	26/107 (24.3)	0.71
APACHE Score	26 (24-27)	29 (27-29.)	0.02
Location of cardiac arrest			
Out-of-hospital no./total-no. (%)	81 (82.7)	89 (83.2)	0.92
In-hospital no./total-no. (%)	17 (17.3)	18 (16.8)	
Cause of cardiac arrest			
AMI-no./total no. (%)	76 (77.6)	77 (72)	0.71
Primary arrhythmia-no./total-no. (%)	16 (16.3)	24 (22.4)	
Respiratory-no./total-no. (%)	2 (2)	2 (1.9)	
Other-no./total no.(%)	4 (4.1)	1 (0.9)	
Time to ROSC (min)	22 (18-30)	19 (12-27.75)	< 0.01
Total epinephrine dose (mg)	3 (2-6)	2.2 (0-5)	< 0.01
Bystander CPR*	19 (24.3)	44 (99)	0.02
Length of ICU stay (days)	16 (13-21)	12 (9-16)	0.04
Time on ventilator (hours)	217 (180-313)	204 (145-243)	0.20

Data are presented as medians (25th and 75th percentiles) or as absolute numbers (relative frequencies). AMI - acute myocardial infarction, APACHE - acute physiology and chronic health evaluation, ROSC - return of spontaneous circulation. * Bystander CPR; data are available from n = 78 in the control group and n = 106 in the hypothermia group.

Neurological outcome was defined at the time of discharge from ICU according to the Pittsburgh cerebral performance category (CPC) [7]. CPC 1 and 2 were classified as a favorable neurological outcome whereas CPC 3, 4 and 5 were regarded as an unfavorable outcome. A follow-up concerning mortality was completed for all patients after 24 months.

The SPSS software (Version 17.0) and Medcalc (Version 11.0) were used for statistical analysis and graphical depiction. Descriptive parameters are given as median and interquartile range (25-75 percentiles). Univariate analysis of differences between hypothermia patients and the control group was performed by using the Mann-Whitney-U test for non-parametric unpaired data. Survival data were analyzed by the Kaplan-Meier method and comparison between groups was performed by the log-rank test. To adjust for confounders a Cox-regression analysis was calculated.

## Results

### Study population

During the observation period, 107 consecutive comatose patients after VF cardiac arrest were admitted to our MICU. The baseline characteristics are given in Table 1. Therapeutic hypothermia was initiated and maintained for 24 hours in all of these patients without any relevant complications. When comparing the hypothermia patients with the historical control group significant differences concerning epinephrine dosage (p < 0.01), time to ROSC (p < 0.01), APACHE score at admission (p = 0.02), rate of bystander CPR (p = 0.020) and length of ICU-stay (LOS; p = 0.040) were found.

### Neurological outcome

Data on neurological outcome of the patient groups at ICU discharge is presented in Table 2. In the hypothermia group 64 patients (59.8%) were discharged with a favorable neurological outcome whereas only 24 patients (24.5%) of the control group had a good neurological outcome. The difference between the groups was statistically highly significant (p < 0.01).

**Table 2:** Neurological outcome of the study population

Neurological outcome	Control	Hypothermia	p-Value
no./total-no. (%)	(n = 98)	(n = 107)	
CPC 1	13 (13.3)	46 (43)	< 0.01
CPC 2	11 (11.2)	18 (16.8)	
CPC 3	10 (10.2)	4 (3.7)	
CPC 4	26 (26.5)	4 (3.7)	
CPC 5	38 (38.8)	34 (31.8)	
CPC 1-2	24 (24.5)	64 (59.8)	
CPC 3-5	74 (75.5)	42 (39.2)	< 0.01

Neurological outcome assessed as cerebral performance category (CPC) at ICU discharge. Data are presented as absolute numbers and relative frequencies.

In contrast CPC 5 was almost equally distributed (hypothermia CPC 5 31.8%, control CPC 5 38.8%).

### 2-year survival

A follow-up concerning mortality was performed after 24 months. Six patients of the hypothermia group and 11 patients of the control group were lost to follow-up. 101 patients treated with therapeutic hypothermia and 87 control patients were included in this analysis.

**Figure 1:**
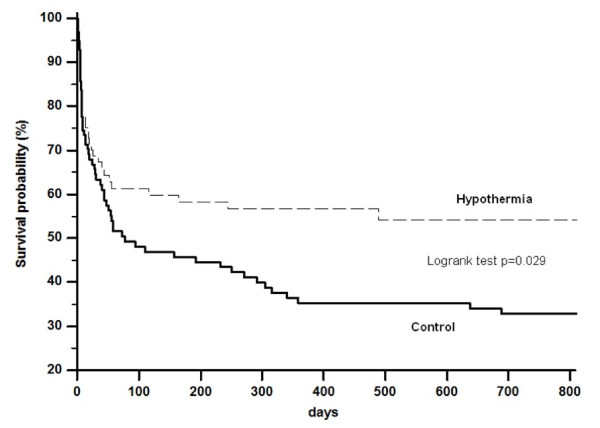
**Kaplan-Meier-survival analysis of both study groups**. A 2 year follow up was available for n = 101 in the hypothermia group and n = 87 in the control group. The difference between the two groups was significant (Logrank test p = 0.029).

The Kaplan-Meier analysis showed a significantly higher 2-year probability of survival in the hypothermia group (hypothermia 55% vs. control 34%; p = 0.029; Figure 1).

The hazard ratio for long-term mortality was calculated with HR = 1.55 (95% CI: 1.04-2.29).

Univariate analysis showed significant differences between the groups for epinephrine dose, time to ROSC, bystander CPR and APACHE-score. Therefore a Cox-regression analysis was performed to adjust for these possible confounders. Hypothermia treatment (p = 0.031) and age (p = 0.013) were the only parameters identified as independent significant predictors for the probability of 24-month survival (Table 3.).

**Table 3:** Cox-regression analysis

Variable	Coefficient	HR	95% CI	p-Value
Epinephrine	0.039	1.04	0.980-1.103	0.198
Time to ROSC	-0.002	1.00	0.977-1.019	0.856
Bystander CPR	0.190	1.21	0.857-1.707	0.280
Hypothermia	-0.403	0.70	0.463-0.963	0.031
Age	0.018	1.02	1.004-1.032	0.013
Gender	-0.076	0.93	0.623-1.378	0.706
APACHE	0.009	1.01	0.986-1.032	0.445

Regression coefficients, HR Hazard ratio, 95% CI confidence intervals, and *P *values of Cox-regression model. Sign (- or +) indicates negative or positive effect on the dependent variable. APACHE II, Acute Physiology and Chronic Health Evaluation II; CI, confidence interval; bystander CPR, bystander cardiopulmonary resuscitation; Time to ROSC, time to return of spontaneous circulation.

## Discussion

In our study we demonstrate a significantly improved 2-year survival of patients treated with mild therapeutic hypothermia after cardiac arrest compared to historical controls. Our findings are in accordance with the HACA trial which revealed a 14% lower mortality rate after 6 months in the hypothermia group [8]. In contrast long-term survival did not differ in a cohort of OHCA patients treated with therapeutic hypothermia observed by Bro-Jeppesen et al. [9]. This may be explained by a remarkably low mortality in the control group. These partly contradictory results emphasize limitations of an observational study design. This also applies to our results, we compared hypothermia patients with historical controls. Therefore it is possible that the improved survival rate is associated with other changes in resuscitation practice as well. For example the rate of successful resuscitation increased significantly between 1992 and 2005 in a large Swedish cohort probably due to an increase in bystander CPR [10]. A similar tendency was observed in our patient groups with significant differences regarding time to ROSC and epinephrine dosages as probably major outcome determinants after cardiac arrest [11]. To adjust for these confounders a Cox-regression model was calculated, revealing hypothermia treatment and age as independent predictors for probability of 24-month survival. Furthermore early cardiac catheterization may have a major impact on outcome of patients resuscitated from VF [12]. Additionally, local treatment protocols may be an influence towards a more sophisticated care of cardiac arrest survivors [13].

We found that significantly more patients were classified CPC 4 in the control group. During the observation period the standard of postresuscitation care has not been changed except for the implementation of the hypothermia treatment protocol. Therefore this remarkable difference in neurological outcome rather reflects an effect of therapeutic hypothermia than posing a bias to statistical analyses.

Furthermore time on ventilator and ICU stay were in part significantly shorter under hypothermia treatment, whereas distribution of mortality at ICU-discharge (CPC 5) was almost identical. Thus the outcome at ICU discharge in both groups was probably not significantly influenced by more early therapy withdrawal in the treatment group. Nevertheless, neurological status may influence the further development and therefore mortality of these patients. It cannot be fully excluded that patients in a good condition are more likely to receive sophisticated medical therapies than patients in persistent coma. This may have also resulted in a higher probability of survival in the treatment group which of course can be only indirectly attributed to hypothermia treatment. Reliable data concerning witnessed arrest and a time delay to defibrillation which could also influence survival are not available unfortunately.

In summary, it is known that the prognosis of patients after out-of-hospital cardiac arrest is similar to that of patients with acute myocardial infarction if they survive until hospital discharge [14]. Our study demonstrates that besides improved neurological outcome the early survival benefit seen with therapeutic hypothermia persists after two years. This should further encourage the implementation of recommendations regarding postresuscitation care to all patients after cardiac arrest suffering from VF and maybe other rhythms as well.

## Conclusion

In conclusion, our data demonstrate that therapeutic hypothermia may be effective in two ways: First of all the neurological outcome at ICU discharge is significantly improved. Furthermore, there is a long lasting benefit concerning probability of survival when therapeutic hypothermia has been applied.

## Abbreviations

AMI: Acute myocardial infarction; APACHE: Acute Physiology and Chronic Health Evaluation; CPC: Cerebral Performance Category; CPR: Cardiopulmonary resuscitation; HACA: Hypothermia after Cardiac Arrest trial; ICU: Intensive care unit; IQR: Interquartile range; OHCA: Out-of-hospital cardiac arrest; VF: Ventricular fibrillation; ROSC: Return of spontaneous circulation.

## Competing interests

The authors declare that they have no competing interests.

## Authors' contributions

CS, JN and DH designed and supervised the study from data acquisition to data analysis. AK and JCS participated in the design of the study, revised the manuscript for important intellectual content and helped to draft the manuscript. All authors have read and approved the final version of the manuscript.

## References

[B1] Castren M Silfvast T Rubertsson S Niskanen M Valsson F Wanscher M Scandinavian clinical practice guidelines for therapeutic hypothermia and post-resuscitation care after cardiac arrest Acta Anaesthesiol Scand 2009 53 280 288 10.1111/j.1399-6576.2008.01881.x 19243313

[B2] Nolan JP Morley PT Hoek TL Hickey RW Therapeutic hypothermia after cardiac arrest. An advisory statement by the Advancement Life support Task Force of the International Liaison committee on Resuscitation Resuscitation 2003 57 231 235 10.1016/S0300-9572(03)00184-9 12858857

[B3] Holzer M Behringer W Schorkhuber W Zeiner A Sterz F Laggner AN Mild hypothermia and outcome after CPR. Hypothermia for Cardiac Arrest (HACA) Study Group Acta Anaesthesiol Scand Suppl 1997 111 55 58 9420956

[B4] Holzer M Bernard SA Hachimi-Idrissi S Roine RO Sterz F Mullner M Hypothermia for neuroprotection after cardiac arrest: systematic review and individual patient data meta-analysis Crit Care Med 2005 33 414 418 10.1097/01.CCM.0000153410.87750.53 15699847

[B5] Nielsen N Hovdenes J Nilsson F Rubertsson S Stammet P Sunde K Outcome, timing and adverse events in therapeutic hypothermia after out-of-hospital cardiac arrest Acta Anaesthesiol Scand 2009 53 926 934 10.1111/j.1399-6576.2009.02021.x 19549271

[B6] Arrich J Zeiner A Sterz F Janata A Uray T Richling N Factors associated with a change in functional outcome between one month and six months after cardiac arrest: a retrospective cohort study Resuscitation 2009 80 876 880 10.1016/j.resuscitation.2009.04.045 19524349

[B7] Jennett B Bond M Assessment of outcome after severe brain damage Lancet 1975 1 480 484 10.1016/S0140-6736(75)92830-5 46957

[B8] Mild therapeutic hypothermia to improve the neurologic outcome after cardiac arrest N Engl J Med 2002 346 549 556 10.1056/NEJMoa012689 11856793

[B9] Bro-Jeppesen J Kjaergaard J Horsted TI Wanscher MC Nielsen SL Rasmussen LS The impact of therapeutic hypothermia on neurological function and quality of life after cardiac arrest Resuscitation 2009 80 171 176 10.1016/j.resuscitation.2008.09.009 19111378

[B10] Hollenberg J Herlitz J Lindqvist J Riva G Bohm K Rosenqvist M Improved survival after out-of-hospital cardiac arrest is associated with an increase in proportion of emergency crew--witnessed cases and bystander cardiopulmonary resuscitation Circulation 2008 118 389 396 10.1161/CIRCULATIONAHA.107.734137 18606920

[B11] Oddo M Ribordy V Feihl F Rossetti AO Schaller MD Chiolero R Early predictors of outcome in comatose survivors of ventricular fibrillation and non-ventricular fibrillation cardiac arrest treated with hypothermia: a prospective study Crit Care Med 2008 36 2296 2301 10.1097/CCM.0b013e3181802599 18664785

[B12] Hosmane VR Mustafa NG Reddy VK Reese CL Disabatino A Kolm P Survival and neurologic recovery in patients with ST-segment elevation myocardial infarction resuscitated from cardiac arrest J Am Coll Cardiol 2009 53 409 415 10.1016/j.jacc.2008.08.076 19179198

[B13] Sunde K Pytte M Jacobsen D Mangschau A Jensen LP Smedsrud C Implementation of a standardised treatment protocol for post resuscitation care after out-of-hospital cardiac arrest Resuscitation 2007 73 29 39 10.1016/j.resuscitation.2006.08.016 17258378

[B14] Engdahl J Bang A Karlson BW Lindqvist J Sjolin M Herlitz J Long-term mortality among patients discharged alive after out-of-hospital cardiac arrest does not differ markedly compared with that of myocardial infarct patients without out-of-hospital cardiac arrest Eur J Emerg Med 2001 8 253 261 10.1097/00063110-200112000-00002 11785590

